# Severity predictors for multisystemic inflammatory syndrome in children after SARS-CoV-2 infection in Vietnam

**DOI:** 10.1038/s41598-024-66891-4

**Published:** 2024-07-09

**Authors:** Dien. M. Tran, Dem. V. Pham, Tung. V. Cao, Canh. N. Hoang, Ha. T. T. Nguyen, Giang. D. Nguyen, Cuong. N. Le, Quan. Q. Thieu, Tuan. A. Ta, Hung. V. Dau, Chi. Q. Le, Quang. H. Le, Nghiem. T. Luong, Mai. T. Tran, Phu. H. Nguyen, Nhung. T. Nguyen, Phuc. H. Phan

**Affiliations:** 1 Surgical Intensive Care Unit, Vietnam National Children’s Hospital, Hanoi, Vietnam; 2 Department of Pediatrics, Faculty of Medicine & Pharmacy, Vietnam National University, Hanoi, Vietnam; 3 Cardiovascular Center, Vietnam National Children’s Hospital, Hanoi, Vietnam; 4 Pediatric Intensive Care Unit, Vietnam National Children’s Hospital, Hanoi, Vietnam; 5 Department of Immunology, Allergy, and Rheumatology, Vietnam National Children’s Hospital, Hanoi, Vietnam; 6 Department of Hematology, Vietnam National Children’s Hospital, Hanoi, Vietnam; 7 Department of Biochemistry, Vietnam National Children’s Hospital, Hanoi, Vietnam; 8 Training and Research Institute for Child Health, Vietnam National Children’s Hospital, Hanoi, Vietnam; 9 Department of Biostatistics, Hanoi University of Public Health, Hanoi, Vietnam

**Keywords:** Multisystemic inflammatory syndrome in children, MIS-C, Severity, Shock, SARS-CoV-2, Diseases, Health care, Medical research, Risk factors, Signs and symptoms

## Abstract

Multisystemic inflammatory syndrome in children (MIS-C) might manifest in a broad spectrum of clinical scenarios, ranging from mild features to multi-organ dysfunction and mortality. However, this novel entity has a heterogenicity of data regarding prognostic factors associated with severe outcomes. The present study aimed to identify independent predictors for severity by using multivariate regression models. A total of 391 patients (255 boys and 136 girls) were admitted to Vietnam National Children’s Hospital from January 2022 to June 2023. The median age was 85 (range: 2–188) months, and only 12 (3.1%) patients had comorbidities. 161 (41.2%) patients required PICU admission, and the median PICU LOS was 4 (2–7) days. We observed independent factors related to PICU admission, including CRP $\ge$ 50 (mg/L) (OR 2.52, 95% CI 1.39–4.56, p = 0.002), albumin $\le$ 30 (g/L) (OR 3.18, 95% CI 1.63–6.02, p = 0.001), absolute lymphocyte count $\le$ 2 (× 10^9^/L) (OR 2.18, 95% CI 1.29–3.71, p = 0.004), ferritin ≥ 300 (ng/mL) (OR 2.35, 95% CI 1.38–4.01), p = 0.002), and LVEF < 60 (%) (OR 2.48, 95% CI 1.28–4.78, p = 0.007). Shock developed in 140 (35.8%) patients, especially for those decreased absolute lymphocyte $\le$ 2 (× 10^9^/L) (OR 2.48, 95% CI 1.10–5.61, p = 0.029), albumin $\le$ 30 (g/L) (OR 2.53, 95% CI 1.22–5.24, p = 0.013), or LVEF < 60 (%) (OR 2.24, 95% CI 1.12–4.51, p = 0.022). In conclusion, our study emphasized that absolute lymphocyte count, serum albumin, CRP, and LVEF were independent predictors for MIS-C severity. Further well-designed investigations are required to validate their efficacy in predicting MIS-C severe cases, especially compared to other parameters. As MIS-C is a new entity and severe courses may progress aggressively, identifying high-risk patients optimizes clinicians' follow-up and management to improve disease outcomes.

## Introduction

From the first reported infection caused by the severe acute respiratory syndrome coronavirus-2 (SARS-CoV-2) in December 2019 in Wuhan City, Hubei Province, China, the epidemic spread worldwide and caused a global health crisis^1^. The WHO has reported 773,119,173 confirmed cases of coronavirus disease in 2019 (COVID-19), including 6,990,067 deaths (https://covid19.who.int/ accessed on 24 December 2023).

At the beginning of the pandemic, reported data suggested that the incidence of SARS-CoV-2 infection occurs more frequently in adults than in children^2,3^. However, the current studies support the remarkable prevalence of asymptomatic infection among children, causing an underestimated burden of COVID-19 in this population^4^. Along with the spiking number of SARS-CoV-2 cases after the new emerging Omicron variant, children potentially remain at risk of diverse post-COVID-19 sequelae afterward that can devastate multiple organs and impact quality of life beyond years^5–7^. Specifically, the most severe complication is multisystemic inflammatory syndrome in children (MIS-C) associated with COVID-19, also called pediatric inflammatory multisystem syndrome temporally associated with SARS-CoV-2 (PIMS-TS). The initial report of MIS-C from the United Kingdom in April 2020 described a cluster of eight children with previous SARS-CoV-2 infection presenting a clinical scenario similar to atypical Kawasaki disease (KD) or toxic shock syndrom^8^. Since then, similar cases have been reported in other parts of the world^9–12^. The theory suggested that the disease is driven by 2–6 weeks post-infectious immune dysregulation; the principal treatment is immunomodulatory therapy combining other supportive therapeutics^13–15^.

MIS-C might present with a heterogeneous spectrum in severity, ranging from mild manifestations to severe complications, including shock, respiratory failure, myocardial involvement, multiorgan dysfunction, coagulopathy, and encephalopathy^11,16–19^. MIS-C pathogenesis is characterized by overwhelming immune system responses, leading to hyper‐inflammation and cytokine storm^8^. This over-inflammatory mechanism is crucial for the explanation of multi-organ damage in MIS-C, in which we hypothesized that the inflammatory markers changes might reflect the MIS-C entity's host response severity and provide clues to identify patients at risk of severity for strict follow-up and timely treatment escalation To date, despite the ongoing efforts to investigate any independent factors that could predict the severity and outcomes of the disease, data on this issue still lacks consistency. Additionally, regardless of the evolution of data from North America and European countries, there are few reports on MIS-C from Asia, leading to an underestimated disease burden in this area^9–12,18,20^. Vietnam has experienced the fourth COVID-19 wave of epidemics, of which the fourth wave from early 2021 had been by far the worst of the pandemic^21,22^. Concomitant with the spiking of COVID-19 infection in children, MIS-C cases increased drastically almost four weeks afterward. However, there is a lack of published data on MIS-C in Vietnam; further investigations are needed. Thus, we conducted this study to identify independent predictors of severity in patients with MIS-C at a tertiary hospital in Vietnam. This available data on epidemiological, clinical, and laboratory characteristics and severe outcomes of MIS-C patients might also enhance pathophysiologic insights and directions for future research.

## Methods

### Study design and participants

This prospective observational study was conducted from January 2022 to June 2023 at Vietnam National Children’s Hospital (VNCH)—the largest referral tertiary children’s hospital of the country. We obtained written informed consent from the parents or legal guardians of all participants, and the study was approved by Vietnam National Children’s Hospital Institutional Review Board #1 (Approval no. VNCH-TRICH-2022-2A). The study was conducted in accordance with the Declaration of Helsinki.

### Case definition

During the study period, we included 391 children aged from 1 month to 18 years old who met the MIS-C case definition of the United States Centers for Disease Control and Prevention (US.CDC)^23^. Accordingly, any illness in a person aged less than 21 years that meets the clinical and the laboratory criteria, which are as follows: (1) fever > 38.0 °C for ≥ 24 h (subjective or documented fever); (2) laboratory evidence of inflammation, including one or more of the followings: high values of C-reactive protein (CRP), erythrocyte sedimentation rate (ESR), fibrinogen, procalcitonin, d-dimer, ferritin, lactic acid dehydrogenase (LDH), or interleukin 6 (IL-6); elevated neutrophils, reduced lymphocytes, and low albumin; (3) evidence of at least two organs injury (respiratory, cardiovascular, hematologic, renal, gastrointestinal, or neurological involvement); (4) current or recent SARS-CoV-2 infection diagnosed by a positive reverse transcription polymerase chain reaction (RT-PCR) or positive serological tests (IgM, IgG or IgA), or exposure to a suspected or confirmed COVID-19 case within the 60 days prior to the onset of symptoms.; (5) no alternative plausible diagnosis and exclusion of any other microbial infections, including bacterial sepsis, staphylococcal or streptococcal shock syndromes, and viral infections associated with myocarditis, such as enterovirus. Patients suspected of or confirmed with other microbial (bacterial, viral, fungal, and parasite) infections were excluded.

### Data acquisition

#### Data on clinical and laboratory characteristics

Data on clinical and demographic characteristics, laboratory and therapeutic variables, and echocardiographic findings within 24 h of admission and daily were routinely collected using standard case report forms (CRFs) (available on the Supplementary 1 for further details). The treatment was supported by strategy guidance from the American College of Rheumatology Clinical Guidance and the Rheumatology Study Group of the Italian Society of Pediatrics^15^.

#### Severe outcomes

There are previous reports in the literature of sudden fatality in MIS-C due to cardiovascular compromise during the hyperinflammatory phase of the illness, mostly related to cardiovascular failure, with hypotension and decreased left ventricle contractility^24^. Additionally, severe involvement of the respiratory, cardiac, neurologic, or renal systems in MIS-C cases was significantly associated with PICU admission^24–26^. Therefore, our variables for severe outcomes in MIS-C were the development of shock within the first 72 h of hospitalization and the requirement of pediatric intensive care unit (PICU) admission. Given the low overall mortality from MIS-C and not all patients admitted to the PICU, death and PICU length of stay were not included as severe outcomes. The shock definition include impaired perfusion signs with or without hypotension, which are a weak and fast pulse, cool peripheries, and a capillary refill time of > 3 s^27^. As there are still no universal criteria for PICU admission, criteria for PICU admission vary by the center worldwide. We defined PICU admission as including one of the following: use of vasopressors, respiratory failure requiring noninvasive or invasive ventilation, abnormal neurological symptoms, evidence of liver or kidney damage, arrhythmia, and presence of myocarditis.

### Statistical analysis

We used a convenient method for determining the sample size of our study. All statistical analyses were performed using STATA software version 17.0 (StataCorp LP, College Station, TX, USA). Categorical variables were described as frequencies and percentages, while continuous variables were described as the median and interquartile range. Results were described as odds ratio (OR) and 95% confidence intervals (CIs). The area under the receiver operating characteristic curve was calculated to compare the predictive performance of the difference biomarkers for MIS-C severity. For each variable, the area under the curve (AUC), 95% CI, and the optimal cutoff point were determined. Variables significantly associated with the outcomes in univariate analyses were selected for multivariate logistic progression to identify independent predictors. A two-sided p-value of less than 0.05 was considered statistically significant.

## Results

### Baseline characteristics of the study population

We analyzed data from 159,410 patients admitted to hospital between January 2022 and June 2023. Of these, 525 patients were reported with MIS-C diagnosis. Among them, 88 patients who unmeet the case definition for MIS-C were excluded. We further excluded 37 patients with insufficient data, 9 patients with microbiologically confirmed infections (5 cases with *Staphylococcus aureus* infections, 2 cases with *Burkholderia pseudomallei* infections, and 2 cases with Adenovirus infections). A total of 391 patients (255 boys and 136 girls) were ultimately recruited in this study (See the flow diagram for study participants in the Supplement 2). The median age was 85 (range 2–188) months and only 14 (3.6%) patients had comorbidities. The frequency of children with a COVID-19 infection history was 310 (79.3%) patients, and the median time since the previous infection prior to symptom onset was 36 (29–47) days. We summarized the epidemographic and clinical parameters of the total study population in Table 1. See Supplement 3 for additional details.

**Table 1:** Characteristics of patients hospitalized with multisystemic inflammatory syndrome in children.

Characteristics	Value (N = 391)
Age (month), median (IQR)	85 (43–114) Min–Max: 2–188
Sex, n (%)	
Male	255 (65.2%)
Female	136 (34.8%)
History of COVID-19 infection	
Yes, n (%)	310 (79.3%)
Time since the previous infection (days)	36 (29–47)
Clinical presentations	
Fever	391 (100%)
Muco-cutaneous involvement, n (%)	359 (91.8%)
Cardiovascular involvement, n (%)	93 (23.8%)
Respiratory distress, n (%)	46 (11.7%)
Gastrointestinal involvement, n (%)	246 (62.9%)
Neurologic involvement, n (%)	79 (20.2%)
Lymphadenopathy, n (%)	162 (41.4%)
Arthralgia, n (%)	12 (3.0%)
Myalgia, n (%)	15 (3.8%)
Outcomes	
Shock, n (%)	140 (35.8%)
PICU admission, n (%)	161 (41.2%)
Coronary dilation, n (%)	101 (25.8%)
PICU LOS (days), median (IQR)	4 (2–7)
Hospital LOS (days), median (IQR)	7 (6–9)
Mortality, n (%)	2 (0.5%)

Data are presented as median (IQR Q1–Q3) or number (%).

*IQR* Interquartile Range, *COVID-19* Coronavirus disease 2019, *PICU* Pediatric Intensitive Care Unit, *LOS* Length of stay.

Among the study population, 161 (41.2%) patients needed PICU admission, and the median PICU LOS was 4 (2–7) days. Shock developed in 140 (35.8%) patients. In our study, we found that 161 patients were admitted to the PICU, with 140 patients experiencing shock that coincided with one or more of the above indications. The remaining 21 patients, without shock, were admitted for respiratory distress (7 cases), seizure (3 cases), evidence of liver or kidney damage (5 cases), arrhythmia (4 cases), and both arrhythmia and respiratory distress (2 cases) (Data not shown). Overall, two deaths (0.5%) occured in our study population.

### Lab findings

Median (IQR) of HGB (g/L), absolute LYM count (× 10^9^/L), PLT count (× 10^9^/L), albumin (g/L), and natremia (mmol/L) were presented in Table 2 (114, IQR 105–122; 1.4, IQR 0.9–2.2; 176, IQR 120–245; 134, IQR 131.1–136, respectively).

**Table 2:** Laboratory characteristics within 24 h of admission.

Parameters	Median (IQR)
Absolute WBC count (× 10^9^/L)	9.7 (7.0–13.3)
Absolute NEU count (× 10^9^/L)	0.7 (0.5–10.4)
Absolute LYM count (× 10^9^/L)	1.4 (0.9–2.2)
Lymphocyte to neutrophil ratio	0.2 (0.1–0.4)
Absolute platelet count (× 10^9^/L)	176 (120–245)
HGB (g/L)	114 (105–122)
CRP (mg/L)	103.2 (59.7–149.5)
PCT (ng/mL)	1.9 (0.6–10.2)
LDH (IU/L)	304.8 (260.7–369.0)
Ferritin (ng/mL)	395 (212–864)
Triglycerid (mmol/L)	1.7 (1.2–3.2)
IL-6 (pg/mL)	65.0 (18.8–186.5)
URE (mg/dL)	3.9 (3.0–5.0)
Creatinin (μmol/L)	48.9 (38.5–57.9)
GOT (IU/L)	36.8 (27.6–61.3)
GPT(IU/L)	35.0 (20.1–60.3)
Glucose (mmol/L)	6.3 (5.4–7.4)
Albumin (g/L)	32.7 (29.8–36.6)
Na (mmol/L)	134 (131.1–136)
K (mmol/L)	3.7 (3.5–4)
d-Dimer (ng/mL fibrinogen equivalent unit)	2168 (1338–4136)
APTT (s)	33.1 (30.0–37.3)
INR	1.2 (1.1–1.3)
Fibrinogen (g/L)	4.5 (3.8–5.2)
Troponin I (ng/mL)	0.03 (0.01–0.11)
ProBNP (pg/mL)	133 (40.7–455)
CK-MB (IU/L)	18.7 (13.5–30.9)
N-antibodies (g/L)	38.5 (19.7–85.5)
S-anibodies (g/L)	64.0 (24.7–223.6)

Data are presented as median (IQR Q1–Q3).

*WBC* white blood cell, *NEU* neutrophil, *LYM* lymphocyte, *HGB* hemoglobin, *PLT* platelet, *CRP* C-reactive protein, *PCT* procalcitonin, *LDH* lactic acid dehydrogenase, *IL-6* interleukin-6, *URE* blood urea nitrogen, *AST* aspartate aminotrans-ferase, *ALT* alanine amino-transferase, *Na* natremia, *K* kalemia, *APTT* activated partial thromboplastin time, *INR* internationalnormalized ratio (INR), *proBNP* pro B-type natriuretic peptide, *CK-MB* creatine phosphokinase-MB, *S-antibodies* antibodies against the spike (S) protein, *N-antibodies* antibodies against the nucleocapsid (N) protein.

**Figure 1:**
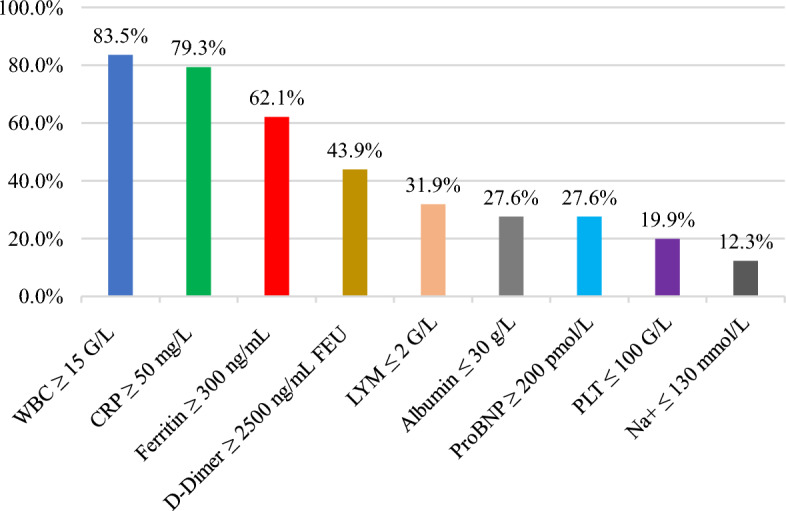
Percentage of patients according to the variable change. *WBC* white blood cell, *CRP* C-reactive protein, *LYM* lymphocyte, *proBNP* pro B-type natriuretic peptide, *PLT* platelet, *Na* natremia.

Figure 1 showed inflammatory markers were often elevated in the study population, including WBC ≥ 15 (× 10^9^/L) (83.5%), CRP ≥ 50 (mg/L) (79.3%), ferritin ≥ 300 (ng/mL) (62.1%), and d-dimer ≥ 2500 (ng/mL) (43.9%). LYM ≤ 2 (× 10^9^/L) and PLT ≤ 100 (× 10^9^/L) were found in 31.9% and 19.9% of patients at admission, respectively. 27.6% and 12.3% of patients presented with elevated cardiac markers and hyponatremia, respectively.

### Prognostic factors of disease severity

#### Shock

Shock developed in 140 (35.8%) patients within the first 72 h after admission. The predictors of shock were identified based on indicators with significant differences, followed by an analysis of the univariate and multivariate logistic regression as presented in Table 3.

**Table 3:** Univariate and multivariate logistic regression for predictors of shock (Linktest p < 0.001 for multivariate logistic regression).

Factors	Univariate regression		Multivariate regression	
	OR (95% CI)	p	OR (95% CI)	p
Fever > 5 (days)	**2.19 (1.29–3.80)**	**0.002**		
Digestive symptoms	**1.62 (1.05–2.52)**	**0.022**		
Age $\ge$ 3 (years)	**3.62 (1.91–7.25)**	** < 0.001**		
WBC ≥ 15 (× 10^9^/L)	**1.96 (1.00–4.00)**	**0.038**		
LYM $\le$ 2 (× 10^9^/L)	**3.51 (2.03–6.21)**	** < 0.001**	**2.48 (1.10–5.61)**	**0.029**
PLT $\le$ 100 (× 10^9^/L)	**2.43 (1.32–4.45)**	**0.002**	1.59 (0.72–3.51)	0.255
Fibrinogen ≥ 5 (g/L)	1.15 (0.69–1.92)	0.569		
d-Dimer $\ge$ 2500 (ng/mL)	**1.61 (1.02–2.56)**	**0.032**	1.16 (0.55–2.29)	0.672
CRP $\ge$ 50 (mg/L)	**2.33 (1.23–4.66)**	**0.006**	1.31 (0.57–3.05)	0.520
Ferritin $\ge$ 300 (ng/mL)	**3.76 (2.04–7.10)**	** < 0.001**		
LDH ≥ 500 (IU/L)	1.62 (0.95–2.77)	0.059		
Albumin $\le$ 30 (g/L)	**3.49 (1.96–6.22)**	** < 0.001**	**2.53 (1.22–5.24)**	**0.013**
LVEF < 60 (%)	**2.68 (1.52–4.66)**	** < 0.001**	**2.24 (1.12–4.51)**	**0.022**
Coronary dilation	0.98 (0.59–1.64)	0.933		
ProBNP $\ge$ 200 (pg/mL)	**2.62 (1.55–4.42)**	** < 0.001**		

Bold font indicates statistical significance.

*WBC* white blood cell, *LYM* lymphocyte, *PLT* platelet, *CRP* C-reactive protein, *LDH* lactic acid dehydrogenase, *LVEF* left ventricular ejection fraction, *proBNP* pro B-type natriuretic peptide.

Absolute LYM count ≤ 2 (× 10^9^/L) (OR 2.48, 95% CI 1.1–5.61, p = 0.029), albumin ≤ 30 (g/L) (OR 2.53, 95% CI 1.22–5.24, p = 0.013), and LVEF < 60 (%) (OR 2.24, 95% CI 1.12–4.51, p = 0.022) were identified to be independently associated with shock on multivariate logistic regression.

#### PICU admission

One hundreds and sixty-one (41.2%) patients required PICU admission, and the median PICU LOS was 4 (2–7) days. Similarly, the predictors of PICU admission were identified based on indicators with significant differences, followed by an analysis of the univariate and multivariate logistic regression as presented in Table 4.

**Table 4:** Univariate and multivariate logistic regression for predictors of pediatric intensive care unit admission (Linktest p < 0.001 for multivariate logistic regression).

Factors	Univariate regression		Multivariate regression	
	OR (95% CI)	p	OR (95% CI)	p
Fever > 5 (days)	0.97 (0.61–1.52)	0.881		
Digestive symptoms	**1.90 (1.25–2.89)**	**0.002**		
Age $\ge$ 3 (years)	**1.78 (1.07–2.98)**	**0.013**	0.97 (0.54–1.75)	0.920
WBC (× 10^9^/L)	1.46 (0.81–2.65)	0.190	1.03 (0.59–1.83)	0.904
LYM $\le$ 2 (× 10^9^/L)	**2.04 (1.89–3.09)**	** < 0.001**	**2.18 (1.29–3.71)**	**0.004**
PLT $\le$ 100 (× 10^9^/L)	**2.85 (1.51–5.53)**	** < 0.001**	0.97 (0.50–1.87)	0.917
Fibrinogen ≥ 5 (g/L)	**1.54 (0.96–2.98)**	**0.047**		
d-Dimer $\ge$ 2500 (ng/mL)	**1.8 (1.10–2.97)**	**0.012**	1.11 (0.69–1.76)	0.670
CRP $\ge$ 50 (mg/L)	**3.49 (1.96–6.27)**	** < 0.001**	**2.52 (1.39–4.56)**	**0.002**
Ferritin $\ge$ 300 (ng/mL)	**2.58 (1.52–4.41)**	**0.001**	**2.35 (1.38–4.01)**	**0.002**
LDH ≥ 500 (IU/L)	1.01 (0.62–1.68)	0.903		
Albumin $\le$ 30 (g/L)	**4.09 (2.10–8.38)**	** < 0.001**	**3.18 (1.63–6.02)**	**0.001**
LVEF < 60 (%)	**3.55 (1.99–6.42)**	**0.001**	**2.48 (1.28–4.78)**	**0.007**
ProBNP $\ge$ 200 (pg/mL)	**2.88 (1.60–5.32)**	**0.011**		

Bold font indicates statistical significance.

*WBC* white blood cell, *LYM* lymphocyte, *PLT* platelet, *CRP* C-reactive protein, *LDH* lactic acid dehydrogenase, *LVEF* left ventricular ejection fraction, *proBNP* pro B-type natriuretic peptide.

Out of these risk factors included in the multivariate model, CRP $\ge$ 50 (mg/L) (OR 2.52, 95%CI 1.39–4.56, p = 0.002), albumin $\le$ 30 (g/L) (OR 3.18, 95% CI 1.63–6.02, p = 0.001), LYM $\le$ 2 (× 10^9^/L) (OR 2.18, 95% CI 1.29–3.71, p = 0.004), ferritin ≥ 300 (ng/mL) (OR 2.35, 95% CI 1.38–4.01), p = 0.002), and LVEF < 60 (%) (OR 2.48, 95% CI 1.28–4.78, p = 0.007) were significantly associated with severe MIS-C requiring PICU admission (Table 4).

Cut-offs of these variables were chosen based on reference values in previous cohort^28–30^.

## Discussion

The present study reported independent predictors of severity in 391 pediatric patients with MIS-C in the tertiary hospital from Vietnam. The results found that lymphocytopenia, hypoalbuminemia, high CRP, and decreased LVEF were independently associated with the risk of severity among children with MIS-C.

According to previous systemic reviews, MIS-C might manifest a broad spectrum of scenarios, ranging from mild features to severe complications, including shock, multiorgan failures, and mortality^5–7^. Regarding clinical presentations, the most frequent symptoms were fever (100%), followed by mucocutaneous symptoms (91.8%), GI involvement (62.9%), lymphadenopathy (41.4%), and cardiovascular findings (23.8%) (Table 1). Ahmed et al. found out that the most common symptoms of MIS-C are fever, GI symptoms (abdominal pain, vomiting, and diarrhea), rash, and conjunctivitis^31^. Similarly, GI symptoms (especially abdominal pain, vomiting, and diarrhea) were also previously considered prevalent symptoms of this novel entity^28,32,33^. The predominance of GI manifestations in children with MIS-C was striking^33^. A plausible pathogenesis might be the overwhelming systemic immune response after COVID-19 infection, including the effect on the digestive system^34^. Besides, by detecting auto-antibodies targeting gastrointestinal antigens in the plasma of MIS-C patients, it was hypothesized that the GI tract may serve as a viral reservoir for continuous exposure to the SARS-CoV-2 SAg-like motif^35^. In the cohort study, 35.8% developed shock, 11.7% had respiratory distress, 41.2% required PICU admission, and 0.5% were death (Table 1). However, the ICU admission rate was less than in cohorts from Hungary (52.6%)^36^ and Turkey (54.5%)^37^. Shock and mortality were documented with a higher proportion in most cohorts from other parts of the world^38,39^. We hypothesized that the variation in shock definition and PICU admission criteria depending on centers, SARS-CoV-2 variants circulating, study sample sizes, and healthcare settings may account for this difference.

Despite being considered as a “new entity” of KD, MIS-C is common in children 9–10 years, whereas classic KD occurs predominantly in children under five years of age^9,18,40^. In line with previous studies, our study identified that the median age group was 7 (3–12) years (Table 1)^41–43^. This point can be explained by the findings that younger children are less likely to contract COVID-19 than older children and adolescents due to several hypotheses related to specific properties of young children, including the lower expression in receptors of angiotensin-converting enzyme 2 (ACE2), cross-protection against SARS-CoV-2 infection, and their immature immune system^4,44^. We found that age ≥ three years was associated with an increased risk of shock (OR 3.62, 95% CI 1.91–7.25, p < 0.001) and PICU admission (OR 1.78, 95% CI 1.07–2.98, p = 0.013) among children with MIS-C, though insignificance of the predictor after adjusting in the multivariate analysis (Tables 3 and 4). Other studies also identified older age as a risk factor for severity in children with MIS-C, despite differences in cut-off point of age. In a previous study on infants with MIS-C, the most severe complications were less common in infants than in the MIS-C cohort in all ages, including cardiovascular involvements, shock, and the ICU admission rate^45^. Similarly, according to Belay et al., compared with patients in the older age categories, patients aged 0 to 4 years had fewer cardiovascular complications and fewer admissions for intensive care^46^. Compared with children aged 0–5 years, the adjusted absolute risk of ICU admission was higher among children aged 6–12 years (43.6% vs 18.4%, adjusted risk difference 25.2%, 95% CI 13.6% to 36.9%) and children aged 13–17 (46.2% v. 18.4%, adjusted risk difference 27.7%, 95% CI 8.3% to 47.2%)^47^. Likewise, the lesser susceptibility to develop severe MIS-C at an early age might not be distinctive in the pediatric population. Recent reviews also highlighted that patients with multisystem inflammatory syndrome in adults (MIS-A) have a higher mortality than MIS-C cases^48^. The overall mortality of MIS-A is between 5 and 7%, whereas that of MIS-C is approximately 1.7%^49^. However, the currently available data is limited, and there is a need for additional studies to enlighten this imparity.

MIS-C pathogenesis is characterized by overwhelming immune system responses, leading to hyper‐inflammation and cytokine storm^8,48,50^. This over-inflammatory mechanism is crucial for explanation of with multi-organ damage in MIS-C, in which biochemical changes could be partly demonstrated by the laboratory inflammatory markers, such as lymphocyte count, CRP, PCT, LDH, ferritin, d-dimer, TNF-α, and IL-6…^20,41,50^. Common lab findings indicative of MIS-C were elevated levels of CRP, ESR, d-dimer, ferritin, PCT, LDH, and a decrease in serum albumin and lymphocyte count as a panel of biomarkers in the existing guidelines^14,15^. The inflammatory markers changes might reflect the MIS-C entity's host response patterns and provide clues to identify patients at risk of severity for strict follow-up and timely treatment escalation. In the present study, absolute LYM count ≤ 2 (× 10^9^/L) were recognized to be independently associated with shock (OR 2.48, 95% CI 1.10–5.61) and PICU admission (OR 2.18, 95% CI 1.29–3.71) on the multivariate model, respectively (Tables 3 and 4). Similarly, an observational study showed lymphopenia was common in classic MIS-C with multiple organ involvement and shock^51^. Bar-Meir et al. reported lymphopenia (lymphocyte count < 1500 µL) was an independent predictor of MIS-C, with an adjusted odds ratio of 24 (95% CI 1.3–326, p = 0.02)^52^. This raised a question about the mechanisms and the role of lymphopenia observed in MIS-C patients. One proposed explanation is the profound lymphopenia caused by marked T cell exhaustion after SARS-CoV-2 infection, which persists for weeks, leading to uncontrolled inflammation and immune dysregulation^53^. We emphasized that further studies are needed to validate whether lymphopenia can predict MIS-C development and severity and whether lymphocyte-targeted therapeutics could improve the disease outcome. We also found that other biomarkers, including d-dimer ≥ 2500 (ng/mL), ferritin ≥ 300 (ng/mL), CRP $\ge$ 50 (mg/L), and albumin ≤ 30 (g/L), significantly related to the severity scenario in the univariate model. However, only albumin ≤ 30 (g/L) was a significant predictor of both shock (OR 2.53, 95% CI 1.22–5.24, p = 0.013) and PICU admission (OR 3.18, 95% CI 1.63–6.02, p = 0.001) (Tables 3 and 4) after adjusted in the multivariate model. The pivotal role of albumin in severity prediction can be explained by an increased capillary permeability and escape of albumin to the interstitial space parallel with the context of the overwhelming inflammation in MIS-C^54^. In a study in 76 MIS-C patients, an albumin level < 33.6 (g/L) was independently related to the risk of PICU admission^26^. Sharon et al. concluded that compared to CRP values with a wide range and high variability throughout the illness, albuminemia is a more reliable and accurate inflammatory marker that needs to be monitored regularly, particularly in critically ill children^55^. Similarly, Abrams J.Y. et al. noted that high ferritin, C-reactive protein, and D-dimer were associated with life-threatening manifestations, including shock and heart depression^56^. A d-dimer > 2568 ng/mL is an early predictor of severe MIS-C requiring ICU admission^57^. The surge of these biomarkers may reflect the presence of the known hyper-inflammatory condition and cytokine storm theory seen in MIS-C; therefore, immunoregulatory agents, such as IVIG, corticosteroids, interleukin blockers, are the mainstays in the treatment of MIS-C^13–15^. To date, IVIG (2 g/kg) plus high dose methylprednisolone (10–30 mg/kg/day) for three consecutive days is the first choice in MIS-C patients with shock, cardiac injury, or neurological damage^15^. The initial guidelines for the MIS-C were extrapolated from KD treatment. However, a more violent inflammatory activation was found in MIS-C, and it raised controversies around whether only IVIG or corticosteroid therapy is sufficient in patients with MIS-C. According to a study comparing efficacy among corticosteroids alone, IVIG alone, and IVIG plus corticosteroids group, there was an insignificant difference in initial responses between patients receiving corticosteroids alone and IVIG plus corticosteroids^58^. However, this outcome might be due to heterogeneous characteristics in disease severity between the groups; further studies on this issue are needed. In addition, the unavailability and high cost of IVIG and biological agents might strain the approach to this therapy, particularly in resource-limited settings. This point partly explains why our study reported that 19.2% received intravenous corticosteroids plus IVIG and only 1.5% received biological therapy (Table 1).

Cardiovascular complications are common manifestations in MIS-C, with an incidence of 40% to 80%^17,46^. Cardiovascular complications in MIS-C might present from mild manifestations to severe ones, such as arrhythmias, pericardial effusion, coronary artery aneurysms, myocarditis, shock, and life-threatening^10,16,17^. Therefore, cardiac markers, including proBNP, NT-proBNP, and troponin, are potential parameters to predict the progression of MIS-C deterioration. Several studies reported elevated BNP or NTpro-BNP, troponin levels, and LVEF related to required ventilation, PICU admission, or mortality^17,59,60^. Similarly, our study showed that proBNP ≥ 200 (pg/mL) and LVEF < 60 (%) were related to an increased risk of shock and PICU admission. However, we noted that LVEF might be more valuable for the severity prediction, with higher odds risk, than the proBNP level (see Tables 3 and 4). We supposed that this could be explained because proBNP peptides are primarily synthesized and upregulated by myocardial stress; therefore, it only reflects indirect cardiac suppression. Additionally, another report noted that there is a delayed improvement of NT-proBNP levels after myocardial function normalization^60^. In the present study, we then assessed the LVEF < 60% as an independent predictor for shock (OR 2.24, 95% CI 1.12–4.51, p = 0.022) and PICU admission (OR 2.48, 95% CI 1.28–4.78, p = 0.007) in the multivariate model (Tables 3 and 4).

Our study had some limitations. First, the data were collected from a single center in a tertiary hospital. Nevertheless, to the best of our knowledge, this paper highlighted the largest-sample study of this novel syndrome published in Vietnam. Second, this study performed the laboratory parameters at a single time and did not evaluate their variations during the subsequent days. However, this may be beneficial as we can eliminate the effects of therapy during treatment. Finally, we only evaluate the risk for short-term outcomes; further, well-designed research is required to define the prognostic factors identifying the long-term effects of MIS-C.

## Conclusions

In conclusion, our study emphasized that absolute lymphocyte count, serum albumin, CRP, and LVEF were independent predictors for MIS-C severity. Further well-designed investigations are required to validate their efficacy in predicting MIS-C severe cases, especially compared to other parameters. As MIS-C is a new entity and severe courses may progress aggressively, identifying high-risk patients optimizes clinicians' follow-up and management to improve disease outcomes.

### Supplementary Information

Supplementary Information 1.

Supplementary Information 2.

Supplementary Information 3.

## Data Availability

The data sets used and analyzed during this study are available from the corresponding author upon reasonable request.

## References

[1] Huang C (2020). Clinical features of patients infected with 2019 novel coronavirus in Wuhan, China. Lancet Lond. Engl..

[2] Sinha IP (2020). COVID-19 infection in children. Lancet Respir. Med..

[3] Viner RM (2021). Susceptibility to SARS-CoV-2 infection among children and adolescents compared with adults. JAMA Pediatr..

[4] Tran DM (2022). High seroprevalence of anti-SARS-CoV-2 antibodies in children in Vietnam: An observational hospital-based study. Pathogens.

[5] Richard V (2023). Impact of the COVID-19 pandemic on children and adolescents: determinants and association with quality of life and mental health: A cross-sectional study. Child Adolesc. Psychiatry Ment. Health.

[6] Seth S, Rashid F, Khera K (2021). An overview of the COVID-19 complications in paediatric population: A pandemic dilemma. Int. J. Clin. Pract..

[7] Kumar P, Jat KR (2023). Post-COVID-19 sequelae in children. Indian J. Pediatr..

[8] Riphagen S, Gomez X, Gonzalez-Martinez C, Wilkinson N, Theocharis P (2020). Hyperinflammatory shock in children during COVID-19 pandemic. Lancet.

[9] Hisamura M, Asai H, Sakata N, Oi H, Taguchi H (2022). Multisystem inflammatory syndrome in children: A case report from Japan. Cureus.

[10] Pavlyshyn H, Slyva V, Dyvonyak O, Horishna I (2021). Multisystem inflammatory syndrome in children (MIS-C) temporally associated with SARS-CoV-2: The first clinical case in Ternopil, Ukraine. Germs.

[11] Cavalcanti A (2022). Paediatric inflammatory multisystem syndrome temporally associated with SARS-CoV-2 (PIMS-TS): A Brazilian cohort. Adv. Rheumatol. Lond. Engl..

[12] Radia T (2021). Multi-system inflammatory syndrome in children & adolescents (MIS-C): A systematic review of clinical features and presentation. Paediatr. Respir. Rev..

[13] Ravichandran S (2021). SARS-CoV-2 immune repertoire in MIS-C and pediatric COVID-19. Nat. Immunol..

[14] Harwood R (2021). A national consensus management pathway for paediatric inflammatory multisystem syndrome temporally associated with COVID-19 (PIMS-TS): Results of a national Delphi process. Lancet Child Adolesc. Health.

[15] Henderson LA (2021). American college of rheumatology clinical guidance for multisystem inflammatory syndrome in children associated with SARS-CoV-2 and hyperinflammation in pediatric COVID-19: Version 2. Arthritis Rheumatol..

[16] Whittaker E (2020). Clinical Characteristics of 58 Children With a pediatric inflammatory multisystem syndrome temporally associated with SARS-CoV-2. JAMA.

[17] Wu EY, Campbell MJ (2021). Cardiac manifestations of multisystem inflammatory syndrome in children (MIS-C) following COVID-19. Curr. Cardiol. Rep..

[18] Sai BVK (2023). Clinical profile and outcome of multisystem inflammatory syndrome in children (MIS-C) associated with COVID-19 infection: A single-center observational study from South India, Egypt. Pediatr. Assoc. Gaz..

[19] Lewis A (2021). Cerebrospinal fluid in COVID-19: A systematic review of the literature. J. Neurol. Sci..

[20] Giacalone M, Scheier E, Shavit I (2021). Multisystem inflammatory syndrome in children (MIS-C): A mini-review. Int. J. Emerg. Med..

[21] Minh LHN, Khoi Quan N, Le TN, Khanh PNQ, Huy NT (2021). COVID-19 timeline of Vietnam: Important milestones through four waves of the pandemic and lesson learned. Front. Public Health.

[22] Hoang VT (2022). Seroprevalence of SARS-CoV-2 among high-density communities and hyper-endemicity of COVID-19 in Vietnam. Trop. Med. Int. Health.

[23] Multisystem Inflammatory Syndrome (MIS). *Centers for Disease Control and Prevention*. https://www.cdc.gov/mis/mis-c/hcp_cstecdc/index.html (2020).

[24] Feleszko W (2023). Pathogenesis, immunology, and immune-targeted management of the multisystem inflammatory syndrome in children (MIS-C) or pediatric inflammatory multisystem syndrome (PIMS): EAACI Position Paper. Pediatr. Allergy Immunol..

[25] Alshehri SS (2024). Characteristics and outcomes of children with severe acute respiratory syndrome coronavirus 2 (SARS-CoV-2) infection admitted to a quaternary hospital: A single-center experience. Cureus.

[26] Haslak F (2021). Clinical features and outcomes of 76 patients with COVID-19-related multi-system inflammatory syndrome in children. Clin. Rheumatol..

[27] Pocket Book of Hospital Care for Children (2013). Guidelines for the Management of Common Childhood Illnesses.

[28] Alam A (2023). Clinical spectrum and prognostic markers of multi-system inflammatory syndrome in children hospitalised in Northern India. Clin. Epidemiol. Glob. Health.

[29] Grama A (2022). Multisystemic inflammatory syndrome in children, a disease with too many faces: A single-center experience. J. Clin. Med..

[30] Al-Ghafry M (2021). Multisystem inflammatory syndrome in children (MIS-C) and the prothrombotic state: Coagulation profiles and rotational thromboelastometry in a MIS-C cohort. J. Thromb. Haemost. JTH.

[31] Ahmed M (2020). Multisystem inflammatory syndrome in children: A systematic review. EClinicalMedicine.

[32] Jiang L (2022). Epidemiology, clinical features, and outcomes of multisystem inflammatory syndrome in children (MIS-C) and adolescents: A live systematic review and meta-analysis. Curr. Pediatr. Rep..

[33] Lo Vecchio A (2021). Factors associated with severe gastrointestinal diagnoses in children with SARS-CoV-2 infection or multisystem inflammatory syndrome. JAMA Netw. Open.

[34] Chen T-H, Kao W-T, Tseng Y-H (2021). Gastrointestinal involvements in children with COVID-related multisystem inflammatory syndrome. Gastroenterology.

[35] Noval Rivas M, Porritt RA, Cheng MH, Bahar I, Arditi M (2022). Multisystem inflammatory syndrome in children and long COVID: The SARS-CoV-2 viral superantigen hypothesis. Front. Immunol..

[36] Varga P (2023). Multicolored MIS-C, a single-centre cohort study. BMC Pediatr..

[37] Sezer M (2022). Multisystem inflammatory syndrome in children: Clinical presentation, management, and short- and long-term outcomes. Clin. Rheumatol..

[38] Lima-Setta F (2020). Multisystem inflammatory syndrome in children (MIS-C) during SARS-CoV-2 pandemic in Brazil: A multicenter, prospective cohort study. J. Pediatr..

[39] Acevedo L (2021). Mortality and clinical characteristics of multisystem inflammatory syndrome in children (MIS-C) associated with covid-19 in critically ill patients: An observational multicenter study (MISCO study). BMC Pediatr..

[40] McCrindle BW (2017). Diagnosis, treatment, and long-term management of Kawasaki disease: A scientific statement for health professionals from the American Heart Association. Circulation.

[41] Hoste L, Van Paemel R, Haerynck F (2021). Multisystem inflammatory syndrome in children related to COVID-19: A systematic review. Eur. J. Pediatr..

[42] Feldstein LR (2020). Multisystem inflammatory syndrome in US children and adolescents. N. Engl. J. Med..

[43] Rhedin S (2022). Risk factors for multisystem inflammatory syndrome in children: A population-based cohort study of over 2 million children. Lancet Reg. Health Eur..

[44] Matucci-Cerinic C (2021). Multisystem inflammatory syndrome in children: Unique disease or part of the Kawasaki disease spectrum?. Front. Pediatr..

[45] Godfred-Cato S (2021). Multisystem inflammatory syndrome in infants <12 months of age, United States, May 2020–January 2021. Pediatr. Infect. Dis. J..

[46] Belay ED (2021). Trends in geographic and temporal distribution of US children with multisystem inflammatory syndrome during the COVID-19 pandemic. JAMA Pediatr..

[47] Merckx J (2022). Predictors of severe illness in children with multisystem inflammatory syndrome after SARS-CoV-2 infection: A multicentre cohort study. Can. Med. Assoc. J..

[48] Kunal S, Ish P, Sakthivel P, Malhotra N, Gupta K (2022). The emerging threat of multisystem inflammatory syndrome in adults (MIS-A) in COVID-19: A systematic review. Heart Lung.

[49] Worku D (2023). Multisystem inflammatory syndrome in adults (MIS-A) and SARS-CoV2: An evolving relationship. BioMed.

[50] Zhao Y, Yin L, Patel J, Tang L, Huang Y (2021). The inflammatory markers of multisystem inflammatory syndrome in children (MIS-C) and adolescents associated with COVID-19: A meta-analysis. J. Med. Virol..

[51] Verdoni L (2020). An outbreak of severe Kawasaki-like disease at the Italian epicentre of the SARS-CoV-2 epidemic: An observational cohort study. Lancet.

[52] Bar-Meir M (2021). Characterizing the differences between multisystem inflammatory syndrome in children and Kawasaki disease. Sci. Rep..

[53] Consiglio CR (2020). The immunology of multisystem inflammatory syndrome in children with COVID-19. Cell.

[54] Papadopoulou-Legbelou K, Kavga M, Desli E, Fotoulaki M (2022). Multisystem inflammatory syndrome in a child with low inflammatory markers, persistent hyponatremia, and natriuresis. Hippokratia.

[55] Sharon B, Nath C (2023). Albumin is a reliable and accurate biomarker for assessing clinical course of multisystem inflammatory syndrome in children. Open Forum Infect. Dis..

[56] Abrams JY (2021). Factors linked to severe outcomes in multisystem inflammatory syndrome in children (MIS-C) in the USA: A retrospective surveillance study. Lancet Child Adolesc. Health.

[57] Avrusin IS (2023). Determination of risk factors for severe life-threatening course of multisystem inflammatory syndrome associated with COVID-19 in children. Children.

[58] Villacis-Nunez DS (2022). Short-term outcomes of corticosteroid monotherapy in multisystem inflammatory syndrome in children. JAMA Pediatr..

[59] Zhao Y, Patel J, Huang Y, Yin L, Tang L (2021). Cardiac markers of multisystem inflammatory syndrome in children (MIS-C) in COVID-19 patients: A meta-analysis. Am. J. Emerg. Med..

[60] Rodriguez-Gonzalez M, Castellano-Martinez A (2022). Age-adjusted NT-proBNP could help in the early identification and follow-up of children at risk for severe multisystem inflammatory syndrome associated with COVID-19 (MIS-C). World J. Clin. Cases.

