# Supplementary material for: Severity predictors for multisystemic inflammatory syndrome in children after SARS-CoV-2 infection in Vietnam
Source: Sci Rep. 2024 Jul 9;14:15810. doi: 10.1038/s41598-024-66891-4 (PMC11233495; doi:10.1038/s41598-024-66891-4)
Supplement: Supplementary file 1 — Supplementary Information 1. [file 41598_2024_66891_MOESM1_ESM.docx]

**SUPPLEMENT 1: Data on clinical and demographic characteristics, laboratory and therapeutic variables**

Demographic variables included age, sex, weight, ethnicity, comorbidities, and history of COVID-19 infection and vaccination. Clinical variables involved systemic manifestations (mucocutaneous, respiratory, cardiovascular, hematologic, renal, gastrointestinal (GI), or neurological involvement). Laboratory variables included white blood cell (WBC) count (×10^9^/L), lymphocyte (LYM) count (×10^9^/L), neutrophil (NEU) count (×10^9^/L), hemoglobin (HGB) (g/L), platelet (PLT) count (×109/L), CRP (mg/L), PCT (ng/mL), LDH (u/L), ferritin (ng/mL), triglycerid (TG), IL-6 (pg/mL), blood urea nitrogen (URE) (mg/dL), creatinine (CRE) (μmol/L), aspartate aminotrans-ferase (AST) (international units [IU]/L), alanine amino-transferase (ALT) (IU/L), glucose (mmol/L), albumin (g/L) protein (g/L), natremia (Na) (mmol/L), kalemia (K) (mmol/L)*,* troponin I (ng/mL), pro B-type natriuretic peptide (proBNP) (pg/mL), CK-MB (U/L), lactate (mmol/L), prothrombin time (PT) (s), internationalnormalized ratio (INR), activated partial thromboplastin time (aPTT) (s), fibrinogen (g/L), and d-dimer (ng/mL fibrinogen equivalent unit), immunoglobulin A (IgA), immunoglobulin M (IgM), immunoglobulin G (IgG), immunoglobulin E (IgE), CD3, CD4, CD8 (/mL), antibodies against the spike (S) protein (S-antibodies) (g/L), antibodies against the nucleocapsid (N) protein (N-antibodies) (g/L), coronary dilation (yes/no), left ventricular ejection fraction (LVEF), and blood culture results. Therapeutic variables included immunoregulators (corticosteroids, intravenous immunoglobulin (IVIG), biological agents), anticoagulants, and antiplatelets. Tests to detect SARS-CoV-2 infection were performed by RT-PCR using oro/nasopharyngeal swabs or tracheal aspirates and/or serology. We used the criteria proposed by the American Heart Association (AHA) to define the presence of coronary artery abnormalities by the Z-score index (i. No involvement: Always <2; ii. Dilation only: 2 to <2.5; iii. Small aneurysm: ≥2.5 to <5; iv. Medium aneurysm: ≥5 to <10, and absolute dimension <8 mm; v. Large or giant aneurysm: ≥10, or absolute dimension ≥8 mm).
