# Supplementary material for: Severity predictors for multisystemic inflammatory syndrome in children after SARS-CoV-2 infection in Vietnam
Source: Sci Rep. 2024 Jul 9;14:15810. doi: 10.1038/s41598-024-66891-4 (PMC11233495; doi:10.1038/s41598-024-66891-4)
Supplement: Supplementary file 2 — Supplementary Information 2. [file 41598_2024_66891_MOESM2_ESM.docx]

**Supplement 2: The flow diagram for study participants**

*VNCH, Vietnam National Children’s Hospital; MIS-C, multisystemic inflammatory syndrome in children; PICU, pediatric intensive care unit*

88 patients did not meet the case definition for MIS-C

437 patients met the case definition for MIS-C

- 37 patients with insufficient data
- 9 patients with microbiologically confirmed infections:
- 5 cases with Staphylococcus aerius infections
- 2 cases with Burkholderia Pseudomallei infections
- 2 cases with Adenovirus infections

.

- 230 never admitted to PICU
- 161 required PICU admission during hospitalization

391 included in the final analysis

p

159,410

atients admitted to VNCH between

Jan 2022 and June 2023

525 patients reported with suspected MIS-C diagnosis
