# Supplementary material for: Severity predictors for multisystemic inflammatory syndrome in children after SARS-CoV-2 infection in Vietnam
Source: Sci Rep. 2024 Jul 9;14:15810. doi: 10.1038/s41598-024-66891-4 (PMC11233495; doi:10.1038/s41598-024-66891-4)
Supplement: Supplementary file 3 — Supplementary Information 3. [file 41598_2024_66891_MOESM3_ESM.docx]

**SUPPLEMENT 3:** **Characteristics of patients hospitalized with multisystemic inflammatory syndrome in children**

| **Characteristics** | **Value (N = 391)** |
| --- | --- |
| Ethnicity, n (%)  Kinh  Others | 379 (96.9%)  12 (3.1%) |
| Comobidities, n (%)  Yes  Cardiovascular disease  Respiratoy disease  Neurological disease  Malignancy  Others | 12 (3.1%)  5 (1.3%)  1 (0.3%)  1 (0.3%)  1 (0.3%)  4 (0.9%) |
| History of COVID-19 vaccination  Yes, n (%) | 14 (3.6%) |
| Clinical presentations  Fever  Yes, n (%)  Duration of fever (days), median (IQR)  Muco-cutaneous involvement, n (%)  Yes  Rash  Conjunctivitis  Cracked lips  Swollen or red hands and feet  Swollen strawberry tongue  Cardiovascular involvement, n (%)  Yes  Arrhythmia  Hypotension  Prolonged refill  Chest pain  Respiratory distress, n (%)  Gastrointestinal involvement, n (%)  Yes  Abdominal pain  Vomiting  Diarrhea  Neurologic involvement, n (%)  Yes  Headache  Seizure  Ageusia  Anosmia  Lymphadenopathy, n (%)  Arthralgia, n (%)  Myalgia, n (%) | 391 (100%)  6 (4 - 7)  359 (91.8%)  297 (75.9%)  296 (75.7%)  134 (34.3%)  52 (13.3%)  53 (13.5%)  93 (23.8%)  78 (19.9%)  53 (13.5%)  28 (7.2%)  26 (6.6%)  46 (11.7%)  246 (62.9%)  157 (40.2%)  150 (38.3%)  115 (29.4%)  79 (20.2%)  75 (19.2%)  6 (1.5%)  2 (0.5%)  1 (0.3%)  162 (41.4%)  12 (3.0%)  15 (3.8%) |
| Treatment, n (%)  CS  CS + IVIG  CS + IVIG + Infliximab  Anticoagulant  Antiplatelet  Hemodialysis  ECMO | 388 (99.2%)  75 (19.2%)  6 (1.5%)  221 (56.5%)  251 (64.2%)  5 (1.3%)  1 (0.3%) |

*Data are presented as median (IQR: Q1–Q3) or number (%).*

*IQR, Interquartile Range; COVID-19, Coronavirus disease 2019; CS, Corticosteroid; IVIG, Intravenous immunoglobulin; ECMO, Extracorporeal membrane oxygenation.*
